# Mesoscale Modeling Study on Mechanical Deterioration of Alkali–Aggregate Reaction-Affected Concrete

**DOI:** 10.3390/ma15113861

**Published:** 2022-05-28

**Authors:** Weijia Wang, Jimin Wang, Jinting Wang, Jinrong He, Jianwen Pan

**Affiliations:** 1 State Key Laboratory of Hydroscience and Engineering, Tsinghua University, Beijing 100084, China; wang-wj21@mails.tsinghua.edu.cn (W.W.); wangjt@tsinghua.edu.cn (J.W.); 2 Yalong River Hydropower Development Co., Ltd., Chengdu 610056, China; wangjimin@ylhdc.com.cn (J.W.); hejinrong@ylhdc.com.cn (J.H.)

**Keywords:** alkali–aggregate reaction, mesoscale model, deterioration, expansion pattern

## Abstract

The alkali–aggregate reaction (AAR) is a harmful chemical reaction that reduces the mechanical properties and weakens the durability of concrete. Different types of activated aggregates may result in various AAR modes, which affect the mechanical deterioration of concrete. In this paper, the aggregate expansion model and the gel pocket model are considered to represent the two well-recognized AAR modes. The mesoscale particle model of concrete was presented to model the AAR expansion process and the splitting tensile behavior of AAR-affected concrete. The numerical results show that different AAR modes have a great influence on the development of AAR in terms of expansion and microcracks and the deterioration of concrete specimens. The AAR mode of the gel pocket model causes slight expansion, but generates microcracks in the concrete at the early stage of AAR. This means there is difficulty in achieving early warning and timely maintenance of AAR-affected concrete structures based on the monitoring expansion. Compared with the aggregate expansion model, more severe cracking can be observed, and a greater loss of tensile strength is achieved at the same AAR expansion in the gel pocket model. AAR modes determine the subsequent reaction process and deterioration, and thus, it is necessary to develop effective detection methods and standards for large concrete projects according to different reactive aggregates.

## 1. Introduction

The alkali–aggregate reaction (AAR) is a harmful chemical reaction that can cause expansion and premature cracking in concrete structures to threaten the safety and durability in the life span. With sufficient moisture, the reactive components in aggregates such as silica can react with the alkaline pore solution in the mortar to form the AAR gel [1]. The gel is a kind of porous material with a high specific surface area and a high hydrophilic property, which will lead to the absorption of water and the expansion of the gel [2]. In the process of AAR, the interior tension is triggered by expansion, and microcracks surrounding the AAR gel are generated. With continuous expansion, microcracks appear increasingly inside the concrete, even expanding to the surface of the concrete structure. In actual projects, the occurrence of AAR inside concrete can give rise to surface map cracking and mechanical deterioration. The persistent expansion and cracking result in the double-negative effect of decreased mechanical properties and increased costs in maintenance and reestablishment [3,4]. Therefore, to better inhibit or reduce the impact of AAR on engineering, it is necessary to deepen the understanding of the AAR mechanism and its deterioration effect.

The expansion and cracking process of AAR-affected concrete involves a wide range of physical processes and scales. There have been many studies to explain the expansion mechanism of AAR-affected concrete based on experiments and theoretical frameworks [5]. Numerical models are also effective as an alternative procedure [6,7,8,9,10]. The macromodel based on phenomenological approaches has the ability to predict the expansion deformation of structures [11,12,13]. In contrast, the meso- or microscale model is better for depth analysis and provides insights into the origins and driving factors of AAR processes [14]. These details are conducive to deepening the understanding of the mechanism of AAR.

The type of alkali-activated minerals results in different AAR modes. In the experimental specimens, the network of microcracks grows as the gel swells, and sometimes, debonding at the interface of the aggregate and the paste can be observed. The assumption of the AAR mode in the mesoscale model determines the process of gel formation and subsequent cracking. It was assumed that AAR mainly occurs on the aggregate surface in some mesoscale models [15,16,17,18]. Bazant and Steffens [19] proposed that the hydrated gel first fills up the micropores near the aggregate surface, and then further formations of gel accumulate the expansion pressure in the interface zone of the aggregate and cement. Finally, the accumulated pressure is released by cracking the cement, but not the aggregate. Romain et al. [20] proposed an approach to explore the formation and structure of gels in solution, and found that the effects of drying and calcification result in crack propagation in the cement. Based on the rigid body spring model, Luo et al. [21] simulated AAR expansion and cracking in concrete specimens with rebar, which provided extrinsic and intrinsic restraints. Pan et al. used the mesoscale model based on the particle discrete element method and AAR aggregate expansion assumption to analyze the reaction mechanism and mechanical property deterioration of AAR-affected concrete [22,23].

Meanwhile, another AAR mode is recognized. The AAR gel has been observed inside aggregates in laboratory experiments [24,25,26,27]. Under the conditions that active reaction sites are inside the aggregate, the alkaline solution needs to gradually penetrate the aggregate to make the AAR occur. Subsequently, during the process of AAR, the gel forms, and the cracks expand from the aggregate into the cement phase. Therefore, a mesoscale model that assumes that the gel forms inside the aggregate has been developed. Dunant et al. [28] presented a two-dimensional model based on a mesostructure to simulate the AAR and gel formation. Cuba et al. [29] developed a finite element model to examine the influence of gel pockets to the mechanical properties of concrete, and found that the density of the gel is the most important factor, instead of the material properties. Miura et al. [30] developed a three-dimensional model to investigate the effect of the expansive site position on AAR-effect cracking propagation and interior stress distribution. Qiu et al. proposed a method to imitate the microstructure of AAR based on relevant images [31], and advanced a 3D reactive transport mesoscopic model based on thermodynamic and kinetic theory to simulate the chemical reaction process [32]. Yang et al. [33] proposed an ASR model to investigate the impacts of temperature and humidity on AAR. In addition, they found that the mechanical deterioration was intensely correlated with heterogeneous expansion and microcracks inside the specimen caused by AAR [34].

The previous study generally investigated the AAR expansion. The different AAR modes may cause varying degrees of deterioration in concrete. This feature has not been touched upon. In this paper, the features of the AAR process and the mechanical deterioration of concrete due to the two well-recognized AAR modes are investigated. The aggregate expansion model and the gel pocket model are established on the basis of the mesoscopic model and the discrete element method to represent the two AAR modes. The reaction process of AAR and the difference in their influence on the mechanical deterioration of concrete are analyzed. First, the mesoscale particle discrete element model of AAR-affected concrete is presented. Then, the strength of the concrete specimen with different AAR expansions is tested by splitting tensile tests based on mesoscale models. Finally, the influence of AAR modes on subsequent reaction processes and the mechanical deterioration of AAR-affected concrete is discussed.

## 2. Mesoscale Modeling Approach

### 2.1. Mesoscale Model of AAR

The mesoscale model of concrete adopted in this study was developed based on two-dimensional mesoscopic particle element theory. It was considered a three-phase material composed of aggregates, mortar, and an interfacial transition zone (ITZ) [35,36,37].

The type of alkali-activated minerals in the aggregate is proven to affect the AAR. During the process of AAR, the gel-formation sites change according to the minerals, which leads to different expansion patterns. In some AAR-affected concrete, AAR gel is observed at the ITZ between the aggregate and cement [19,26]. This expansion pattern was considered the uniform expansion of aggregates and was modeled using the aggregate expansion model [22,23] in this study. By means of the thermodynamic method, the aggregate expands by changing the radius of the particles and updating the strength of the parallel bond between the particles in the aggregate expansion model. The other expansion pattern corresponds to the circumstance that gel is produced inside the aggregate. It was simulated using the gel pocket model, and gel particles were randomly distributed inside the aggregate to simulate the AAR expansion [28].

The AAR expansion of concrete was first modeled based on different AAR expansion patterns (aggregate expansion model and gel pocket model). Then, splitting tensile tests were carried out to examine the deterioration of the AAR-affected concrete specimens using numerical simulations.

### 2.2. Mesoscale Particle Model of Concrete Samples

Based on the Laguerre tessellation [38], the discrete particle model of concrete samples with different aggregate gradations was generated (aggregate radius: 5–20 mm, 20–40 mm). The specimens were 100 mm × 100 mm in size and composed of round particles with an average diameter of 1.4 mm. The distribution curve of particle size obeyed a normal distribution and the ratio of the largest particle radius to the smallest particle radius was selected as 1.5. The volume proportion of aggregates in the samples is approximately 60% [23].

For each type of concrete, three specimens with different aggregate distributions were established. The aggregate expansion model and gel pocket model were considered for AAR simulation on the same aggregate distribution of concrete specimens. In the gel pocket model, the ratio of gel particles to aggregate particles was set to 5%, 1.5%, and 0.5%. For simplicity, the specimens with the AAR mode of the aggregate expansion model were named RC*d-i*, and specimens with the gel pocket model were named GC*d-i-j*, where *d* (*d* = 40, 20) denotes the maximum aggregate size (MAS), *i* (*i* = 1, 2, 3) denotes the specimen number, and *j* (*j* = 5%, 1.5%, 0.5%) denotes the gel ratio.

**Figure 1:**
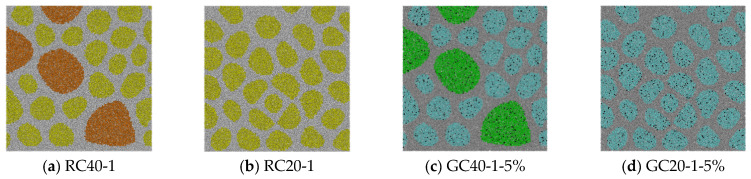

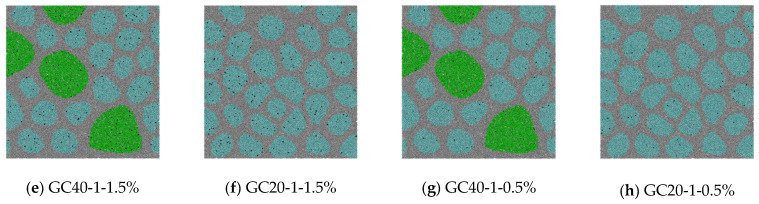
Concrete specimens with different gradations and different expansion modes. (**a**,**b**): Yellow—aggregates with MAS = 20 mm, orange—aggregate with MAS = 40 mm, light gray—mortar. (**c**–**h**): Cyan—aggregate with MAS = 20 mm, green—aggregate with MAS = 40 mm, gray—mortar, black—gel pocket.

Figure 1 shows the mesoscale particle model with different expansion modes and different gel proportions. Table 1 reflects the proportions of aggregates in specimens.

## 3. Results

### 3.1. AAR Process of Concrete

**Figure 2:**
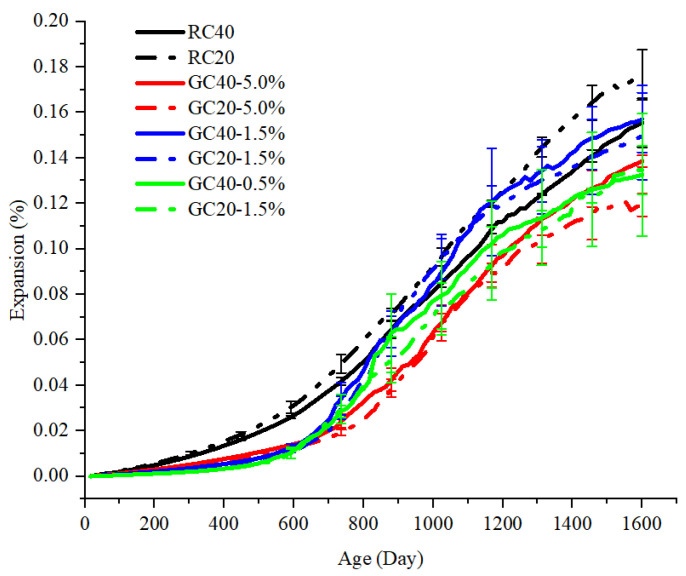
Expansion of concrete specimens with different AAR modes over time.

In the mesoscale numerical simulation, the free deformations of the sample in the x- and y-directions were measured. The average of the deformations in the two directions was considered as the expansion of AAR. Figure 2 shows the expansion of the concrete specimen with different AAR modes. The expansion curves of RC40 and RC20 exhibit a similar trend of expansion over time. The expansion shows an “S”-type curve that is similar to the experimental results [5]. The concrete with greater aggregates gains a smaller AAR expansion. The expansion of the GC specimens, in which the AAR mode was modeled with the gel pocket model, is comparable with the RC specimens at the late stage of AAR, despite being much smaller at the early stage. This phenomenon shows that the gel pocket type of the AAR mode generates a lower expansion rate at the early stage, but a higher expansion rate thereafter. The AAR is well hidden at the early ages and has been reacting for a long period when the monitoring expansion indicates the AAR of the concrete structures. This is harmful to concrete structures that cannot receive timely maintenance. On the other hand, the length of the error bars of the GC specimens is longer than that of the RC specimens, indicating that the distributed gel pockets result in greater discreteness. It can be seen that there is no significant difference in the curves of the different aggregate gradations. This demonstrates that the influence of aggregate gradations on expansion in the GC specimen is less than that in the RC specimen.

**Figure 3:**
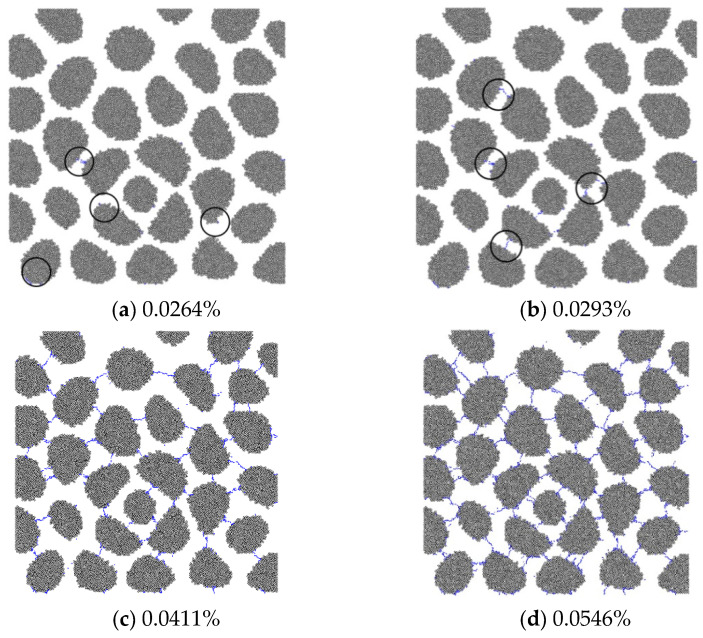
AAR-induced microcracks in RC20-1 as AAR expands.

The process of AAR-induced microcrack initiation and propagation can be seen clearly in the mesoscale model. Figure 3 shows the development of microcracks in RC20 specimens. When the AAR expansion reaches 0.0264%, microcracks first appear in the ITZ around the aggregate. With increasing expansion, new microcracks expand along the previous initial microcracks and propagate in the mortar. As the expansion reaches 0.0411%, the microcracks in the ITZ and mortar are connected, forming a microcrack net in the specimen. It is noted that microcracks do not occur in the aggregates, and all the aggregates remain intact after AAR.

**Figure 4:**
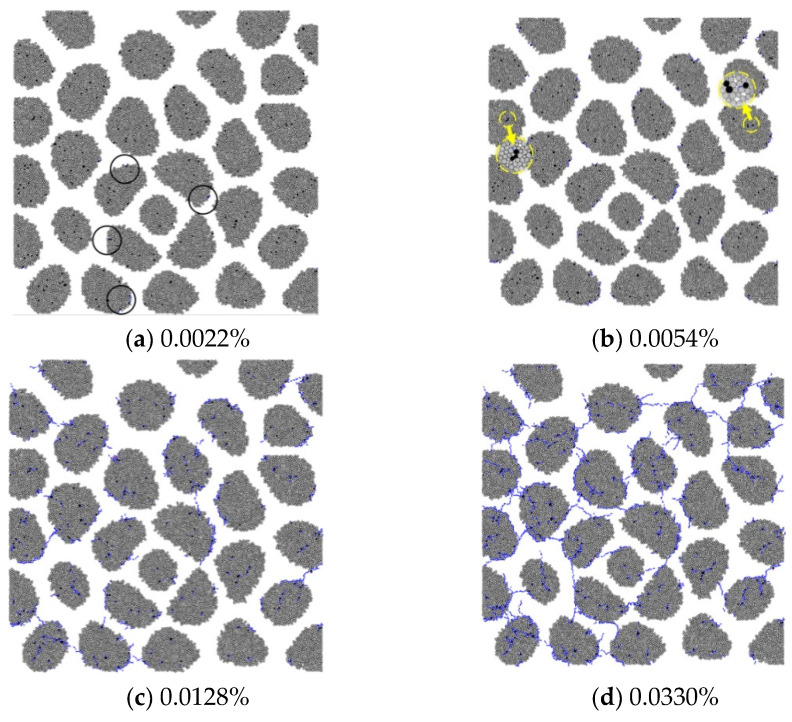
AAR-induced microcracks in GC20-1-1.5%.

Figure 4 shows the microcracks of the GC20-1-1.5% specimen. The development of the microcracks is significantly distinguished from that of the RC specimens due to the different AAR modes. The microcracks initiate at the ITZ and inside the aggregates. As the expansion increases, these microcracks develop and split the aggregates and then extend to the mortar. The microcrack network is formed when the expansion reaches 0.0330%.

**Figure 5:**
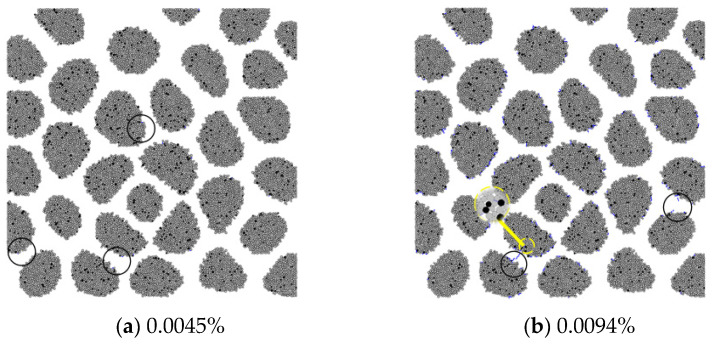

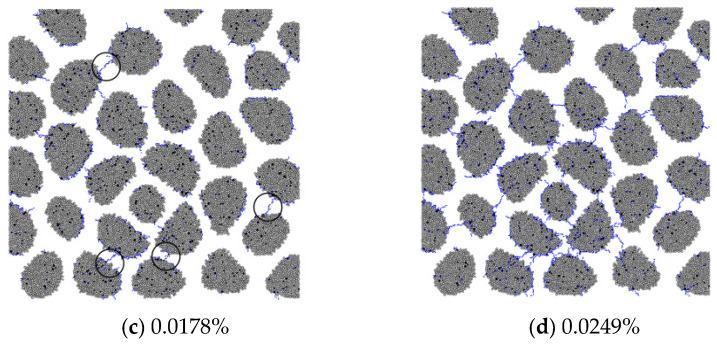
AAR-induced microcracks in GC20-1-5%.

**Figure 6:**
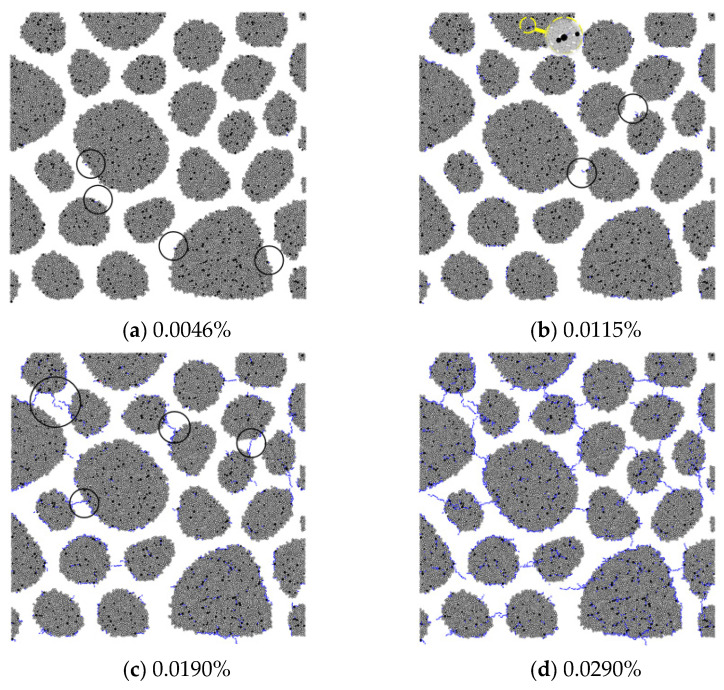
AAR-induced microcracks in GC40-1-5%.

Considering the different proportions of AAR gel distributed in the specimens, Figure 5 shows the development of microcracks of the GC20-1-5% specimen for comparison. The microcracks exhibit a propagation process similar to that in the GC20-1-1.5% specimen. Furthermore, the influence of aggregate size on AAR-induced cracking is examined. Figure 6 shows the development of microcracks of the GC40-1-5% specimen, and it appears similar to the GC20-1-5% specimen. The results imply little influence of the gel proportion and aggregate size on the AAR-induced cracking characteristics.

**Figure 7:**
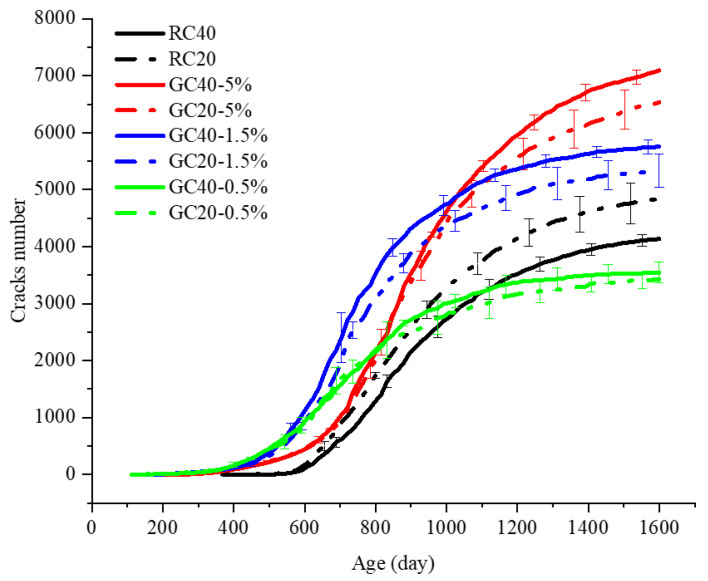
AAR-induced microcrack number over time.

Figure 7 shows the microcrack number in concrete specimens with the development of AAR. The microcrack number was counted according to the bond breakage of the mesoscale particle model. The average of the three samples is considered, and the error bar represents the discreteness among the three samples. Compared to the RC40 specimen, the RC20 specimen has a larger number of microcracks. This phenomenon may be attributed to the larger specific surface area in smaller aggregates. In GC specimens with the same gel proportion, the one with larger aggregates gains more microcracks. This is attributed to the lower strength between the larger aggregate and the mortar. Meanwhile, on the basis of the same aggregate distribution and gradations, the microcrack number of the specimen with a higher gel proportion generates more microcracks. On the other hand, compared to the RC specimen, the GC specimen shows cracks earlier, which is contrary to the AAR expansion process.

### 3.2. Deterioration of the Concrete Specimen

There are few previous studies on the degradation of mechanical properties due to different AAR modes. Herein, only the results from the numerical simulation were examined. Different degrees of AAR lead to different microcrack damage of the specimens. The tensile strength obtained by the disc splitting tensile test was used as an index to evaluate the AAR-induced deterioration of concrete. After the splitting tensile test, the microcrack distribution on the failure mode of the specimens was conductive to analyzing the effects of AAR.

**Figure 8:**
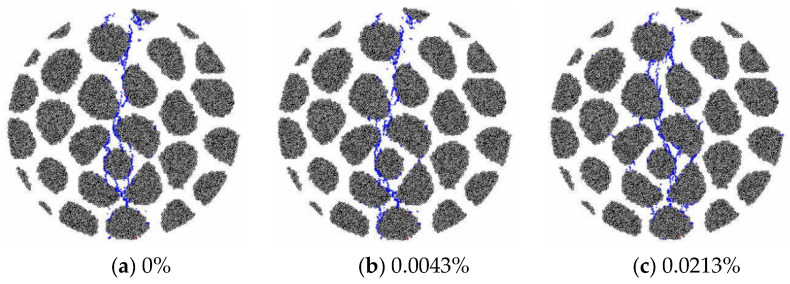

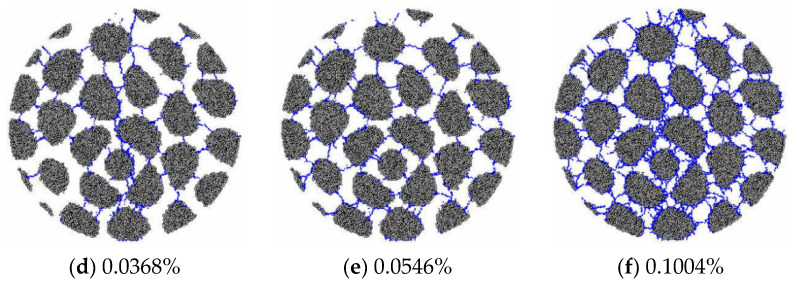
Microcrack morphology in the failure state of RC20 specimen.

**Figure 9:**
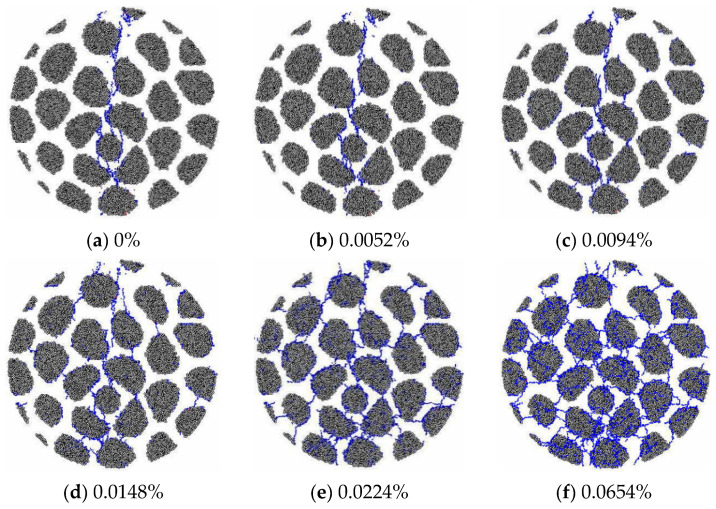
Microcrack morphology in the failure state of the specimen GC20-5%.

The microcrack morphology in the tensile failure state of the RC20 specimen with different expansions is shown in Figure 8. There are no obvious differences in cracking patterns caused by AAR before the expansion reaches 0.02%. The higher degree of AAR further influences the case of damage inside the specimens, which leads to the smeared distribution of microcracks. The microcrack distribution of the GC20-5% specimen, by contrast, is presented in Figure 9. Microcracks are localized in the center of the disc and cause failure of the specimen when the expansion is small, which is similar to the phenomenon observed for the RC20 specimen. As the AAR expansion increases, the microcracks generated by the mechanical loading interact with the AAR-induced microcracks, and the failure mode exhibits a smeared distribution of microcracks instead of a localized distribution. There is a significant difference from the RC20 specimen. Obvious microcracks are distributed in the aggregates and break the aggregates in GC20-5%.

**Figure 10:**
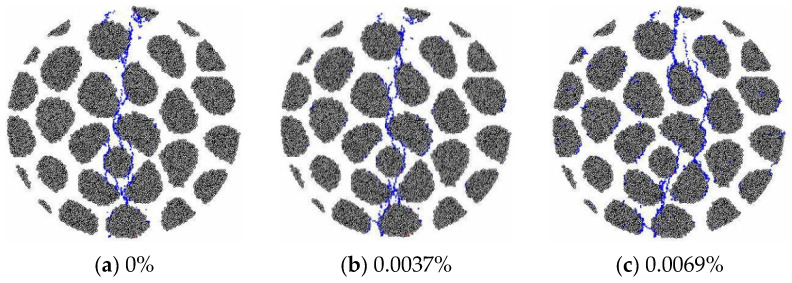

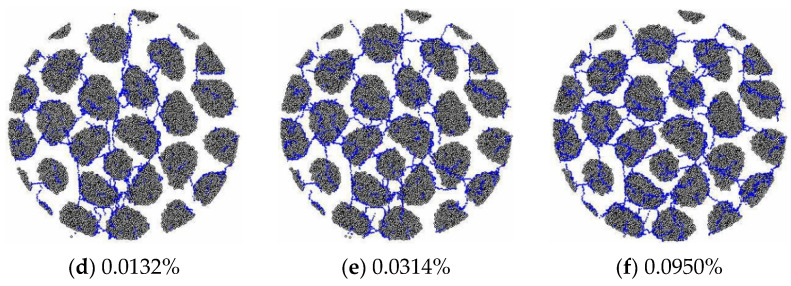
Microcrack morphology in the failure state of the specimen GC20-1.5%.

The microcrack morphology in the tensile failure state of specimen RC20 with different expansions is shown in Figure 10. It is compared with that of GC20-5%. The same tendency of microcrack distribution reveals that the variation in gel proportion affects the splitting failure mode.

**Figure 11:**
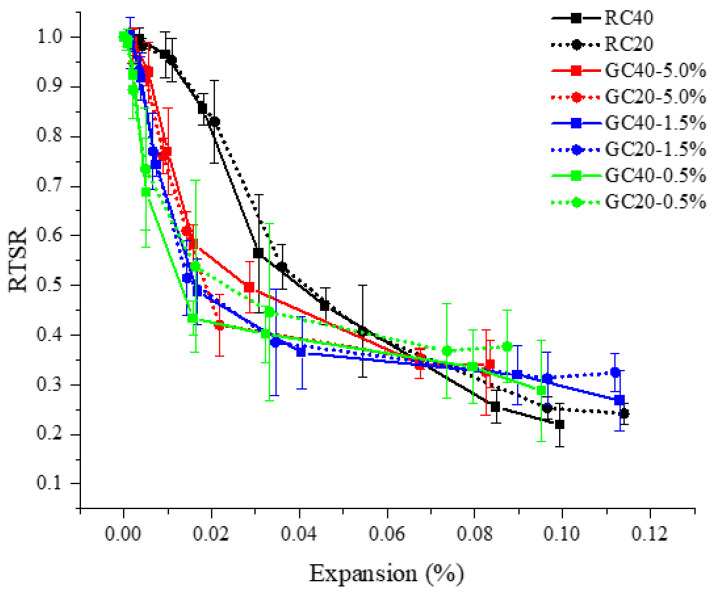
Declining trend of RTSR with AAR expansion of the specimens.

The residual tensile strength ratio (RTSR) was defined as an indicator to measure the degradation of the splitting tensile strength, which can be calculated as the ratio of the tensile splitting strength of the specimen conducted to AAR to the strength of the specimen without AAR. Figure 11 illustrates the deterioration progress of AAR-affected concrete specimens. As the AAR expansion increases, the mechanical properties of the specimen obviously decrease. The aggregate gradation has no significant effects on the tendency of RTSR. Compared to RC specimens, GC specimens have a deeply declining tendency in the RTSR when AAR expansion is smaller than 0.068%. It is suggested that the expansion of distributed gel particles leads to earlier cracking and subsequent deterioration. Drawing a comparison between the GC specimens with different gel proportions, the RTSR has a similar decreasing tendency with the AAR expansion. This implies that the amount of distributed gel particles has a slight effect on the RTSR as long as the same AAR expansion occurs.

## 4. Conclusions

In this paper, the process of microcrack propagation and the deterioration of concrete affected by AAR was investigated based on the mesoscale particle model. Two different AAR modes, i.e., the aggregate expansion model and the gel pocket model, were considered, and their influence on the AAR expansion and splitting tensile strength was analyzed. The main conclusions are drawn as follows.

1. There are differences in the microcrack development process of concrete specimens with different AAR modes. When the initial microcracks appear in the ITZ between the aggregate and mortar, the new microcracks tend to expand to the mortar region along the initial microcracks in the aggregate expansion model, and the new microcracks tend to appear around the aggregate continually in the gel pocket model. The most distinguishing difference between the two AAR modes is that aggregates remain intact in the aggregate expansion model, and obvious cracks appear in aggregates in the gel pocket model.

2. The relationship between AAR expansion and aggregate gradation is different in various AAR modes. In the aggregate expansion model, the specimen with larger aggregates has a lower expansion. In the gel pocket model, the expansion of the specimen does not depend on the aggregate gradation.

3. AAR modes have no significant change in the failure mode with AAR expansion. The failure mode of the concrete specimen with slight AAR expansion exhibits localized cracking, while a high degree of AAR leads to the smeared cracking failure mode of the concrete specimen subjected to the splitting tensile test.

4. The decay speed of the mechanical property is different in various AAR modes. The splitting tensile strength of concrete significantly decreases as AAR expansion increases. The degradation of tensile strength is much greater in the concrete specimen at the AAR expansion less than 0.06% when the AAR mode follows the gel pocket model compared with the aggregate expansion model.

5. In the same AAR expansion, different AAR modes would lead to varying degrees of structural damage that are not necessarily detected. The AAR mode of the gel pocket model causes slight expansion but generates microcracks in the concrete at the early stage of AAR. This means there is difficulty in achieving early warning and timely maintenance of AAR-affected concrete structures based on the monitoring expansion. However, the monitoring expansion is effective for concrete structures with the AAR mode of the aggregate expansion model.

## Figures and Tables

**Table 1:** Aggregate proportions of concrete specimens.

No.	Aggregate Proportion (%)	
	5~20 mm	20~40 mm
RC40-1	35.1	26.5
RC40-2	30.4	31.6
RC40-3	35.3	22.2
RC20-1	55.1	—
RC20-2	49.1	—
RC20-3	56.3	—
GC40-1	35.1	26.5
GC40-2	30.4	31.6
GC40-3	35.3	22.2
GC20-1	55.1	—
GC20-2	49.1	—
GC20-3	56.3	—

## Data Availability

Not applicable.
